# Exploring Health-Promoting Attributes of Plant Proteins as a Functional Ingredient for the Food Sector: A Systematic Review of Human Interventional Studies

**DOI:** 10.3390/nu12082291

**Published:** 2020-07-30

**Authors:** Marta Lonnie, Ieva Laurie, Madeleine Myers, Graham Horgan, Wendy R. Russell, Alexandra M. Johnstone

**Affiliations:** 1 Rowett Institute, School of Medicine, Medical Sciences and Nutrition, University of Aberdeen, Ashgrove Road West, Aberdeen AB25 2ZD, UK; mswmyers@gmail.com (M.M.); g.horgan@abdn.ac.uk (G.H.); w.russell@abdn.ac.uk (W.R.R.); alex.johnstone@abdn.ac.uk (A.M.J.); 2 Department of Human Nutrition, Faculty of Food Science, University of Warmia and Mazury in Olsztyn, 10-718 Olsztyn, Poland; 3 Tate & Lyle, 1 Kingsway, London WC2B 6AT, UK; ieva.laurie@tateandlyle.com

**Keywords:** dietary protein, plant protein, sustainable protein, cardiovascular, metabolic, blood pressure, weight loss, diabetes, muscle

## Abstract

The potential beneficial effects of plant-based diets on human health have been extensively studied. However, the evidence regarding the health effects of extracted plant-based proteins as functional ingredients, other than soya, is scarce. The aim of this review was to compile evidence on the effects of extracted protein from a wide range of traditional and novel plant sources on glycemic responses, appetite, body weight, metabolic, cardiovascular and muscle health. A comprehensive search of PubMed, EMBASE and The Cochrane Central Register of Controlled Trials (CENTRAL) was conducted through 23 and 27 March 2020 for randomized controlled trials that featured any of the following 18 plant protein sources: alfalfa, duckweed, buckwheat, chickpea, fava bean, hemp, lentil, lupin, mushroom, oat, pea, potato, pumpkin, quinoa, rapeseed, rice, sacha inchi, sunflower. Only interventions that investigated concentrated, isolated or hydrolysed forms of dietary protein were included. Searched health outcome measures were: change in blood glucose, insulin, satiety hormones concentration, subjective assessment of appetite/satiety, change in blood lipids concentration, blood pressure, body weight and muscle health parameters. Acute and sub-chronic studies were considered for inclusion. Applying the Preferred Reporting Items for Systematic Reviews and Meta-Analyses (PRISMA) approach we identified 1190 records. Twenty-six studies met the inclusion criteria. Plant protein sources used in interventions were most often pea (*n* = 16), followed by lupin (*n* = 4), fava bean (*n* = 2), rice (*n* = 2), oat (*n* = 2), hemp (*n* = 2) and lentil (*n* = 1). Satiety and postprandial glycemic response were the most frequently reported health outcomes (*n* = 18), followed by blood lipids (*n* = 6), muscle health (*n* = 5), body weight (*n* = 5) and blood pressure (*n* = 4). No studies on the remaining plant proteins in the extracted form were identified through the search. Most studies confirmed the health-promoting effect of identified extracted plant protein sources across glycemic, appetite, cardiovascular and muscular outcomes when compared to baseline or non-protein control. However, the current evidence is still not sufficient to formulate explicit dietary recommendations. In general, the effects of plant protein were comparable (but not superior) to protein originating from animals. This is still a promising finding, suggesting that the desired health effects can be achieved with more sustainable, plant alternatives. More methodologically homogenous research is needed to formulate and validate evidence-based health claims for plant protein ingredients. The relevance of these findings are discussed for the food sector with supporting market trends.

## 1. Introduction

With the expansive population, the demand for dietary protein is increasing globally [1]. The main challenges for the food supply systems are not only to meet this growing demand, but also to provide protein that would be of high quality, health-promoting, and sustainable. Although protein from meat, poultry, fish, dairy and eggs have optimal amino acid composition and high digestibility, the majority of large-scale animal protein production practices raise environmental concerns [2,3]. Moreover, dietary patterns characterised by frequent consumption of meat—the leading source of protein in the Western world [4]—have been associated with increased risk of cardiovascular disease, metabolic syndrome, some forms of cancers and premature mortality [5,6,7,8,9,10,11,12,13,14,15,16]. Consumer interest in plant-based eating has also been steadily increasing globally. European consumers are choosing plant-based products mainly for health and environmental reasons [17]. Consumers report plant-based eating as being considered healthy and a quarter of UK consumers even report feeling guilty about eating animal-based products [18]. Interestingly, parents are also looking for plant-based and meat-free alternatives for their children, with over half reporting that (in line with the flexitarian approach) their children are having a meat-free dinner at home at least once a week [19].

Ingredient suppliers are responding to consumer trends and food manufacturers’ needs by seeking to produce ingredients with improved cost, nutrition, sensory and stability profiles [17]. Ongoing efforts are being made to find ‘new’ and affordable sources of plant protein, including exploring the possibility of utilising waste streams (e.g., fruit pomace as a by-product of juice, wine or jam processing, composed of unused peel, pulp, and seeds) [20]. One of the sustainable sources of dietary proteins are plants, in particular pulses, oil-producing seeds, cereals, nuts, and vegetables [21]. Novel sources include aquatic plants (algae or duckweed) and fungi; historically (sensu amplo) organisms belonging to the plant kingdom [22]. Health promoting attributes of plant-based diets have been extensively studied, especially in the context of obesity, cardiovascular diseases, diabetes, and hypertension [23,24,25,26,27]. In addition, there is an increasing interest in the effects of high-protein foods on appetite control, weight management and muscle health [28,29,30,31,32]. To date, the majority of dietary interventions focused on whole foods, flours or powdered forms of selected plants, with the predominance of soya as a high-protein (approximately 36 g/per 100 g dry weight) [33], nutritionally balanced plant representatives [34]. However, in addition to protein, plants contain a vast array of diverse components, e.g., fibre, starch and bioactive compounds. The use of unprocessed or mildly processed plant protein sources (like whole foods or flour) in dietary interventions can, therefore, be challenging to control for other confounders. Subsequently, it may lead to pre-emptive conclusions, attributing the health benefits exclusively to protein, rather than the food matrix.

Since people consume foods and not nutrients, the approach to study the effects of whole foods on health is entirely justified. Each food has its specific matrix, described as the complex assembly of nutrients and non-nutrients interacting physically and chemically, that influences the release, mass transfer, accessibility, digestibility, and stability of many food compounds [35]. However, studying the effects of individual components in isolation can be equally important. First, investigating the effects of extracted protein on health may improve the understanding of underlying physiological mechanisms and identify components that play a crucial, health-promoting role, for example; certain amino acids and bioactive peptides. Secondly, since functional foods are becoming increasingly popular among health-conscious consumers, extracted forms of plant protein (added to snacks or beverages) can be expected to become a large part of daily protein intake. Hence, from the legislative point of view, it would be important to assess health promoting attributes of the purified plant protein ingredients entering the market.

### Study Aim

Extracted protein can be in the form of concentrates (usually above 70% protein on a moisture-free basis), isolates (>90%) and hydrolysates (mixtures of polypeptides, oligopeptides and amino acids that are manufactured from protein sources using partial hydrolysis) [36]. To our knowledge no previous studies have systematically reviewed the evidence relating to the individual effects of extracted plant protein—other than soya—on health. The aim of this review was to identify evidence on the effects of extracted protein from a wide range of conventional and novel plant sources on glycemic response, appetite, body weight, blood lipids concentration, blood pressure and muscle health and explore the findings with relevance for the food sector.

## 2. Materials and Methods

The present systematic review was conducted in accordance with the Cochrane handbook for systematic reviews of interventions [37]. The results were reported following the PRISMA statement [38]. A protocol for this systematic review was registered on PROSPERO [CRD42020181225] prior to data extraction.

### 2.1. Search Strategy and Data Sources

The review question was structured using the following elements—Population of interest (P); Intervention (I); Comparisons (C); Outcome (O); Study type (S)—namely, the PICOS format [39]. To identify studies relevant to guiding questions, the search strategy was constructed in consultation with the librarian. The description of PICOS elements is displayed in Table 1.

The initial strategy was developed for PubMed and was then adjusted for two additional electronic databases; EMBASE and Cochrane Central Register of Controlled Trials. Searches employed the National Library of Medicine’s Medical Subject Headings (MeSH terms) nomenclature for searches in PubMed and Emtree terms nomenclature for searches in EMBASE. In addition, keywords were searched in singular and plural forms (if applicable) in titles and abstracts fields. Search string (PubMED) is presented in Supplementary Material.

### 2.2. Study Eligibility Criteria

All searches were limited to interventional, human studies with no restrictions regarding sex, age (>18) or health status. Studies on wholefoods or flours consumed alone, or as a part of a diet were excluded. Only studies that strictly investigated the effect of purified preparations of plant protein were considered, in line with the functional ingredient project’s brief. Hence, the interventions included in this review were limited to: concentrates, isolates and hydrolysates. Studies that investigated plant protein only as an addition (or enrichment) to the test food were not included to the final summary, to avoid the confounding effects of other protein sources or the effect of protein blend matrix. Only studies were plant protein ingredient was the only (virtual) source of protein were considered. Studies of genetically modified organisms or plants with altered amino acid profiles were not considered. Studies investigating the effects of medical/prescription food and those referring to weaning food or infant formula were not included. Reviews, editorials, letters to the editor or other publications not reporting primary research findings were not considered. Conference abstracts and posters were considered if all data of interest was reported. Only studies with full text availability and published in English language were reviewed.

### 2.3. Study Selection Process

A comprehensive search was performed during 23–27 March 2020 in the PubMed, EMBASE and Cochrane Library databases. Two investigators (ML and MM) independently screened titles and abstracts for article relevance, based on the agreed title and abstract screening exclusion criteria. Discrepancies were resolved through discussion, with the involvement of a third researcher from the team (AJ), to increase sensitivity of the screening process. A meta-analysis was considered, however, due to heterogeneity of studies (mainly referring to study design, protein doses, forms, carriers and comparators), it was decided that the review would be of a qualitative nature.

## 3. Results

### 3.1. Search Results

**Figure 1:**
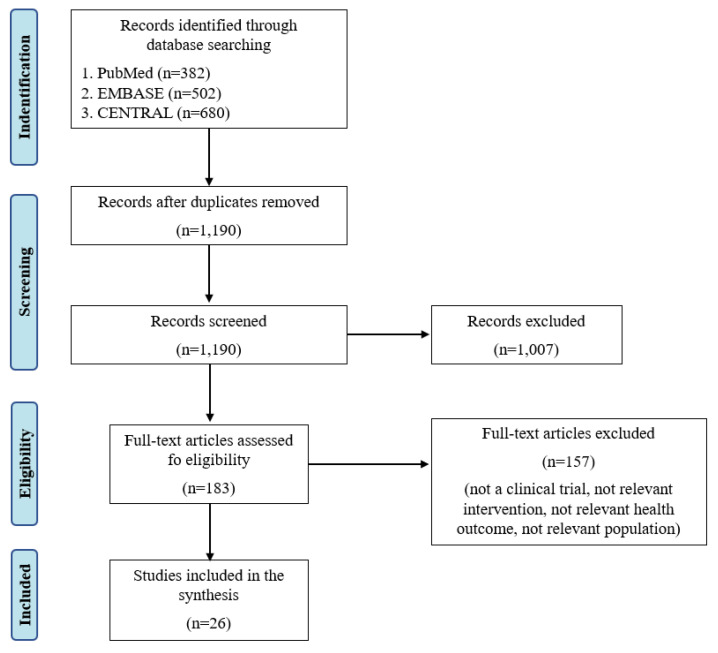
Preferred Reporting Items for Systematic Reviews and Meta-Analyses (PRISMA) flow-chart.

The search yielded a total of 1564 citations. After duplicate removal, 1190 abstracts were screened by two reviewers for relevance to search criteria. In total, 183 references met the predefined criteria for inclusion in the full-text assessment. Based on the full-text reviews, 26 references were thus considered for inclusion in the review (Figure 1).

### 3.2. Trials’ Characteristics

Plant protein sources used most often in interventions were pea (*n* = 16), followed by lupin (*n* = 4), fava bean (*n* = 2), rice (*n* = 2), oat (*n* = 2), hemp (*n* = 2) and lentil (*n* = 1). Satiety and postprandial glycemic response was the most frequently studied health outcome (*n* = 18), followed by blood lipids (*n* = 6), muscle health (*n* = 5), body weight (*n* = 5) and blood pressure (*n* = 4). The summary of included studies is presented in Table 2. The interventions incorporated proteins in a form of isolates in 18 studies, concentrates in seven and hydrolysed forms of protein in four. Dietary daily doses of protein ranged from 10 g [40,41] to 70 g [42] of plant proteins (excluding one study on pea protein hydrolysate—administered in a significantly lower dose) [43]. The average length of the trials depended on the health outcome studied. An acute design (effects after single or multiple administration within 24 h) was used in studies investigating postprandial glycemia and appetite ratings. A sub-chronic design (RCT duration usually up to 3 months) was employed in studies investigating the effects on blood lipid concentration, blood pressure and muscle health, with the longest intervention lasting for 12 weeks [44]. In the majority of studies, proteins were administered in a liquid form (*n* = 17) (Table 3, Table 4, Table 5, Table 6 and Table 7).

### 3.3. Satiety, Glycemic and Insulinemic Responses

The majority of trials identified in the search referred to the effects of plant proteins on postprandial concentrations of blood glucose, insulin and appetite regulating hormones [40,41,42,45,49,50,51,52,53,54,57,58,59,60,61,62,63,65] (Table 3). The most frequently reported proteins in this context were pea proteins [40,41,45,49,50,51,52,54,57,58,61,62,63], followed by fava bean [42,59], rice [51,62], lupin [61,65], oats [62], lentil [53] and hemp [60]. The dosage of protein was reported in two formats: absolute values (g/day) or in g per kg of body weight. The single dose of protein used in interventions ranged from 10 g [40,41] to 50 g for females and 70 g for males [42]. The majority of studies used the acute design, while only three studies were of longer duration [42,61,65], with the longest study lasting for six weeks [65]. Authors used different types of protein carriers with majority being in a liquid form: drinks (smoothies, shakes, added to soup) [41,45,49,50,51,52,53,54,58,59,60,62,63], while others were served protein in bars or integrated into a protein-manipulated meal [40,42,57,61,65]. There was a large heterogeneity in terms of the comparator foods and controls. The most commonly used comparator were milk protein (whey or casein), or other plant proteins (soya), while the control was often a test food without protein or a non-protein ingredient (e.g., maltodextrin).

The majority of studies reported a beneficial effect of extracted plant protein on regulating postprandial glycemia, in comparison to either, placebo or other proteins, including soya; however, the effects varied by study design. When compared to animal protein the effects of plant protein were comparable (no significant between-treatment effects) rather than superior [45,49,57,58,63,65]. In terms of protein form, one study reported that concentrated and isolated forms had a superior effect to that of the flours [59].

In terms of appetite and satiety, the differences between dietary interventions were often not significant, although some studies suggested a trend towards a satiating effect of pea protein [45,52]. Subsequent food intake was investigated in eight studies [40,41,45,52,53,54,57,58], seven investigating pea protein intake and one lentil concentrate and isolate [53]. In four studies, food intake was significantly lower when compared to control [41,45,54,58] while another other four studies did not find significant differences [40,52,53,57]. A detailed description is provided in Table 3.

### 3.4. Blood Lipids

The effects of extracted plant protein on blood lipid concentrations were studied in six trials [42,46,47,61,62,65] (Table 4). Three studies investigated the effects of lupin [46,47,65], one of fava bean [42], one of oat, rice and pea concentrates [62] and one on pea and lupin isolates combined with fibre fractions [61]. Although the latter study was investigating the effect of both, protein and fibre, it was decided that due to the limited evidence in this subject, the results of the study will be included in this narrative review.

The study by Weisse et al. [65] enrolled 43 participants with moderate hypercholesterolemia. A dose of 35 g of lupin protein isolate (LPI) was administered daily over a period of six weeks. Proteins were incorporated into snack bars, which participants were instructed to consume twice a day. When compared to baseline, a significant decrease in total cholesterol was observed in both intervention groups (LPI and casein—as a comparator), although the change did not differ between the groups. Also, compared to baseline, the decrease in LDL cholesterol was observed only in the LPI group, while the decrease in HDL was observed only in the casein group. Moreover, in the casein group, a decrease was also observed within groups for triglyceride concentrations. The only between-treatments difference was observed in LDL:HDL ratio (decrease in the LPI group) [65]. The results of this study need to be carefully compared to other studies within the context of this health outcome, since lupin protein were not the sole source of protein in the tested product. Treatment snack bars also contained wheat and hazelnut flours, in smaller quantities. Although concern was raised whether this study meets the inclusion criteria, due to limited evidence and the fact, that lupin protein were a predominant source of protein, it was decided to include these results in the final synthesis.

Bahr et al. conducted two trials with LPIs [46,47]. In the first study [46] with 33 hypercholesterolemic subjects, a significant reduction was observed in LDL cholesterol (a decrease of −0.26 mmol/L) and LD:HDL ratio (−0.29) after four weeks of 25 g of daily LPI administration. In comparison, the reduction obtained with milk protein was only observed for LDL cholesterol levels, but not the LD:HDL ratio. The effects of both interventions disappeared in week eight. As suggested by the authors, this could be a result of lower compliance to the dietary intervention [46].

The subsequent intervention from Bahr et al. [47] was altered, by incorporating LPI into a mixed diet (in the previous study, LPI was mixed with water). In total, 72 hypercholesterolemic subjects took part in the study. Over the period of 28 days, participants were receiving 25 g of LPI daily; the dose was spread throughout the day and added to bread, rolls, sausage, spread. At the end of the intervention, subjects receiving LPI had significantly lower total cholesterol (−0.28 mmol/L), LDL (−0.15) and HDL (−0.05) when compared to baseline. Between treatments, significant differences were observed in terms of total cholesterol (lower in the milk protein with arginine group vs. milk protein) and LDL (lower in the LPI group vs. milk protein). Importantly, the changes were more profound in subjects with severe hypercholesterolemia. Since the decrease in total cholesterol was observed in the milk protein intervention supplemented with arginine, but not in the unsupplemented milk protein group, it was suggested, that arginine (relatively high in lupin) may be responsible for the decrease in blood lipid concentrations [47].

A small study in eight subjects (seven cholesterolemic, one healthy) investigated the effects of fava bean concentrate [42]. The intervention lasted 18 days, in which participants were administered isocaloric diets with protein accounting for 15% of daily energy intake (fava bean concentrate or egg-white as a comparator, accounted for 11% of daily protein). When compared to baseline, plasma total cholesterol decreased in both diets, although the decrease was significant only in the egg-white intervention. Interestingly, the concentration of LDL cholesterol decreased in the egg intervention, while the HDL decreased with fava beans. No changes were observed in triglyceride concentrations [42].

Also, Tan et al. [62] included plasma triglyceride level as one of the outcome variables, although the blood lipid profile was not the primary outcome. In this study, serum triglyceride excursions and iAUC did not differ between the test beverages, made with oat, rice and pea protein concentrates [62].

Lastly, the effects of lupin and pea protein isolates were investigated with combinations of various fibre fractions, in hypercholesterolemic subjects [61]. A total of 175 subjects were randomized into seven intervention groups. Participants were instructed to consume two protein bars a day over a period of 28 days; protein intake from the bars accounted for 34.6 g/day. A significant reduction in total cholesterol concentrations was observed in the lupin, casein and pea protein groups. Pea protein had an effect on blood lipids only if combined with oat fibre or pectin (decrease in total cholesterol and LDL). No changes were observed in terms of triglicerides and HDL levels [61].

### 3.5. Blood Pressure

Blood pressure was investigated in four studies: two studies tested the effect of pea protein (hydrolysate and isolate) [43,63] and two of lupin isolate [46,47] (Table 4). The sub-chronic study on pea proteins conducted in a sample of seven hypertensive participants revealed a significant reduction in systolic blood pressure (SBP) of 5 and 6 mmHg after two and three weeks of the trial respectively [43]. Since protein in this study was in a hydrolysed form, the doses were as little as 1.5 and 3 g per day, spread over three meals (0.5 or 1 g per meal). Only the higher dose of pea protein hydrolysate (PPH) had a significant blood pressure lowering effect when compared to the placebo.

Postprandial blood pressure was investigated in an acute study with 48 healthy participants [63]. The authors compared the effects of four different protein interventions: milk protein, egg-white protein, pea protein and protein mixture (milk, egg, pea and soya protein). Pea protein isolate (PPI) was administered in a dose of 0.6 g per kg of body weight and consumed mixed with water once a day. When compared to baseline, only the pea protein meal significantly reduced DBP. The ingestion of the pea-protein meal also induced the highest NOx (S-nitrosothiols) response, which is suggested to be attributed to higher arginine content [63].

In the trial with the lupin isolate, a daily dose of 25 g was administered over eight weeks to 33 hypercholesterolemic subjects [46]. In comparison to baseline, lupin protein significantly reduced SBP, DBP and pulse at rest (by 13.6 mmHg, 7.5 mmHg and 4.0 min^−1^, respectively) at week eight of the intervention. A comparative intervention with milk protein used in this study had only observed a significant reduction in SPB, but not DBP or pulse. Blood pressure was also one of the secondary outcomes in another study by Bahr et al. [47], in which LPI was incorporated into food products. However, no effects were observed when compared to the baseline or comparative intervention (milk protein). Since the dose of protein was the same in both studies, it could be that the form of protein administration had an effect (drink vs. protein integrated into foods), with non-significant effect observed in the latter form.

### 3.6. Muscle Health

Muscle health was a subject of five plant protein trials. Two studies investigated the effect of pea protein [44,48], one of rice protein [55], one of oat protein isolate [64] and one of hemp protein concentrate [56]. In the pea protein study, all subjects enrolled were males, aged 18−35 years old [44]. Apart from dietary intervention, participants underwent a course of resistant training on upper limb muscles. The results revealed that muscle thickness increased in the whey and pea protein isolate groups (+15.3% and +13.4% vs. +10.7% in the placebo group) after 12 weeks of supplementation with 25 g of protein consumed twice a day. In the sensitivity analysis (with the weakest participants), the muscle thickness was greater in a pea protein isolate group (+20.2%) than in the whey protein group (15.6%), which suggests that pea protein might be preferential to individuals starting or returning to training [44].

Pea proteins were also discussed by Banaszek et al. [48], who compared the effects of 24 g of pea protein isolate and whey proteins, ingested twice a day, over an eight-week of high-intensity functional training (HIFT) program. By contrast with a study by Babault et al. [44], the sample included men (n = 8) and women (n = 7), with the average age of 39 years in both groups. No differences between the interventions were observed in terms of any outcomes investigated; body composition, muscle thickness, force production or workout performance, implying that pea protein may be perceived as an adequate substitute for whey protein [48].

A higher dose of protein isolate was administered in the rice protein isolate study, in which 24 young men ingested a 48 g dose of either rice protein isolate or whey protein isolate, immediately after the high volume resistance training session, over the course of eight weeks [55]. Both interventions proved to be successful in increasing lean body mass, muscle mass, strength and power, however, no difference was detected between the groups. Although the study is lacking an intervention group with a lower dose of protein administered, the authors suggested that comparable effects to whey protein were achieved due to a large dose of protein administered in this study [55].

Also Kaviani et al. [56] have tested a larger dose (60 g) of protein powder in resistant training among trained individuals. Hemp concentrate was compared the effect of soya protein over the course of eight weeks of resistance training in a mixed-sex group. In this study, increased strength and muscle thickness was observed in females, but not males, in comparison to the soya intervention [56].

The effects of oat protein were studied in a context of muscle damage, inflammation and performance recovery [64]. Sixteen young, untrained men were recruited for the trial. Over the course of 19 days, participants ingested oat protein isolate twice a day; each dose accounted for 12.5 g protein. On the 15th day of the trial, participants were subjected to a downhill treadmill run to induce the delayed onset of muscle soreness (DOMS) and associated damage. The results showed a significant improvement in all measured outcomes; skeletal muscle damage, aseptic inflammation and muscle functioning, when compared to the placebo [64]. The placebo used was maltodextrin, hence it cannot be concluded whether the effect obtained with oat protein is comparable to that of animal protein. However, this was the first study which was investigating the impact of extracted oat protein intake during a muscle damaging exercise in untrained individuals.

### 3.7. Body Weight

Although change in body weight was not a primary outcome of identified trials, five studies provided data on body weigh at baseline and at the end of intervention [42,46,47,48,55]. Two studies investigated the effect of lupin [46,47] while the other studies investigated pea [48], fava bean [42] and rice [55]. All studies used milk protein as a comparator [46,47,48,55] with the exception of one study, which used egg-white protein [42]. In comparison to baseline, Bahr et al. [46] in a trial on lupin isolate reported a slight increase in body weight, BMI, and body fat in both, lupin and milk protein groups. Apart from an increase in waist circumference (in milk protein group vs. lupin group) in a consecutive study by Bahr et al. [47], none of the studies detected significant differences between the interventions.

### 3.8. Risk of Bias and Quality of the Studies

Risk of bias assessment is particularly needed when clinical recommendations are formulated as an outcome of the review. This was not the case in our review. Due to the limited number of studies and their heterogeneity the results from the identified studies could not be pooled together into meta-analysis. Instead, the findings are presented in a narrative and exploratory way, focusing on identifying research gaps. As outlined in the study protocol, it was intended to screen identified studies against the Revised Cochrane Risk-of-bias Tool for Randomized Trials (RoB 2) [66]. However, due to the nature of studies identified the relevance of the tool’s assessment domains was limited. In general, risk of bias due to deviations from intended interventions, bias due to missing outcome data and bias in the measurement of the outcome can be assessed as low to moderate. The comparison of potential bias due to the effect of adhering to intervention was difficult across the studies, because of the mixed-type of study designs. While some studies were sub-chronic, others applied the acute design—the dropouts from the latter type of studies are uncommon due to the usually less than 24h duration. The measurements of the outcomes were based on objective standardised clinical procedures with numerical values as results, hence the risk of bias in measurement of the outcome can also be considered as low. Given the small number of studies the assessment of publication bias was not performed. Potential bias could stem from the randomisation process, blinding, and from the selection of the reported results. Also, by including abstracts (full-versions of articles have not yet been published) [53,56,58,60], there is a risk that the available data were not sufficient to assess the quality of the evidence in those studies. Overall, we did not identify evidence for significant risk of bias or quality concerns for most of the studies. Nevertheless, the findings synthesised in this review should be interpreted with caution.

## 4. Discussion

Data on the health-promoting attributes of non-soya, plant-based proteins in the form of concentrates, isolates or hydrolysates is limited. The conducted search identified randomized trials on pea, lupin, fava bean, rice, hemp, oat and lentil protein. No studies that met the inclusion criteria were found describing the effects of alfalfa, duckweed, buckwheat, chickpea, mushroom, oat, potato, pumpkin, quinoa, rapeseed, sacha inchi or sunflower.

Postprandial glycemic response and appetite were most frequently studied health outcomes. The majority of studies suggested a beneficial effect of extracted plant proteins on regulating postprandial glycemia, in comparison to controls and other protein sources. The interventions lacked homogeneity (mainly in terms of doses and form, e.g., liquid or solid, as well as uniform comparator or placebo), hence a quantitative analysis could not be performed. However, the evidence points towards direction, that investigated plant proteins had a similar, but not a superior effect to that from animal protein (mainly milk proteins, like casein and whey). It has been suggested that the effect of dietary protein ingestion on regulating postprandial glucose and insulin is attributed to the branched-chain amino acids (BCAA)—amino acids that are essential for protein synthesis, but also take part in key metabolic processes (these include: leucine, valine and isoleucine) [67]. In general, legumes contain comparable amounts of these amino acids to animal sources, which may explain no significant effects between treatments, although the concentration of BCAA in blood tend to be higher in individuals on animal protein diet, when compared to the plant one [68]. However, relatively large doses of protein used in identified studies may have exceeded the minimal required threshold for those amino acids [69], hence no significant differences were observed between different protein treatments.

As indicated earlier, the use of different protein carriers was one of the barriers in performing direct comparisons, despite a reasonable number of studies in the context of this health outcome. The majority of studies used a liquid as a carrier, with few studies incorporating protein into solid foods. While it is relatively easy to add a concentrated form of protein into a shake, juice or smoothie (the given source was the only source of protein), the addition of protein to a solid food usually requires an additional filler, such as wheat flour, which is also a source of additional protein. Although this is a commonly used and understandable study protocol, since proteins are not consumed in isolation and are designed to be a potential functional ingredient, it was also one of the reasons why some valuable findings could not be included in the analysis [70,71,72]. In those studies authors investigated the effects of fava bean protein concentrate and isolate [70,71], and pea protein isolate [72], added to pasta, crackers and habitual diet (respectively). The results revealed that the addition of fava bean protein to pasta reduced postprandial glycaemia and appetite, increased PYY and C-peptide responses, however, with significant effects on plasma insulin or GLP-1 [70]. Similarly, Fabek et al. [71] found that the addition of fava bean protein to crackers induced lower postprandial blood glucose (when compared to wheat control), suggesting that the changes were more significant for the isolated form. These studies did not use an animal source of protein as a comparator. In contrast, Markova et al. [72] explored the endocrine responses of plant protein diet in comparison to animal protein in patients with type 2 diabetes. The results of the study revealed, that the diet enriched with pea protein isolate stipulated higher glucagon increase that the diet enriched with casein, and subsequently required higher insulin response. The authors suggested, that the this effect may have been caused by slower uptake and digestion rate of casein in comparison to the pea protein [72].

These above studies are a valuable contribution to the body of evidence, however it would be difficult to compare those effects quantitatively, due to large variability in treatment protocols; particularly: more complex nutritional composition of the test product, usually another source of protein (e.g., wheat)—often in different proportions, and use of more complex processing methods in food preparation. All these factors may have an effect on the glycemic responses, e.g., a particular food matrix, effects of protein blend, or thermal processing of protein. Therefore, if the aim of the study is to investigate and compare the isolated effects of various plant protein sources it would be useful if a non-protein carrier was used as a control, and minimal processing was applied prior to protein ingestion. Also, future studies would benefit from the consistent inclusion of animal protein as a comparator, to ease between-sources comparisons and facilitate formulation of dietary recommendation.

Studies investigating the effects of extracted plant proteins on blood lipid concentration indicated a positive effect on the reduction of cholesterol fractions in hypercholesterolemic subjects. Only one study [42] featuring fava beans confirmed that the lipid lowering effect was greater in a group with the animal protein intervention (egg-white). Lupin was featured in four out of five studies on blood lipids [46,47,61,65]. The lipid-lowering effect was stronger in participants with a higher blood lipid concentration at baseline [47]. The observed blood lipid lowering effects following lupin protein ingestion may be attributed to the lysine to arginine ratio. As observed in a previous animal study, low lysine to arginine ratio may contribute to reducing cardiovascular risks [73].

By contrast, a long-term study (16 weeks) on lupin kernel flour (as a novel ingredient) did not confirm the beneficial effect of an ad libitum enriched lupin flour diet on blood lipids, nor other cardiometabolic factors (body weight, glucose, insulin, plasma leptin) [74]. The authors speculated that lack of significant change could be explained by similar composition of dietary treatments (only a modest difference between the groups in protein intake), structural characteristics of the fibre in the lupin kernel flour (mainly insoluble) or the fact that flour was incorporated into the daily diet (rather than the intake of isolated fibre) [74]. Furthermore, no physiological effect could be attributed to the low content of isoflavones in lupin beans. The underlying mechanisms, however, require further investigation. Despite the promising results, it needs to be highlighted that lupin is a recognised allergen, hence the use of this plant protein source as a functional ingredient is limited.

Blood pressure lowering effects were investigated in four studies [43,46,47,63]. Pea protein and lupin isolate proved to be effective in reducing blood pressure (BP) in three studies [43,46,63], although one study on lupin did not detect significant changes in systolic and diastolic BP in comparison to baseline nor between interventions [47]. Interestingly, the study that used a hydrolysed form of pea protein, achieved a decrease in BP, which was comparable to a single dose of antihypertensive medication suggesting that dietary intervention might be a promising alternative to pharmacological treatment [43]. However, the dose of protein used in this study was very low (1.5–3 g/day), hence the intervention may be perceived as more medicinal, rather than dietary.

Several studies reported the effects of lupin flour on blood pressure. In a long-term RCT, Belski et al. [75] found, that although lupin flour enriched foods (bread, biscuits and pasta) were not effective in enhancing or improving weight loss, positive effects were observed in blood pressure after 12 months of intervention. However, these effects were attributed to substituting carbohydrates (control) with both protein and fibre. By contrast, in a more recent study Ward at al. [76] found that lupin-enriched foods (breakfast cereal, bread, pasta and bread crumbs) consumed over the course of eight weeks (at 45 g/day dose of lupin; 12 g lupin protein and 10 g lupin fibre) had no effect on systolic and diastolic BP in diabetic patients. To our knowledge, no other extracted plant proteins were investigated in the context of BP. It would be interesting to investigate if the BP lowering effect can be achieved with other plant protein sources, and whether the magnitude of this effect will depend on the form used (unprocessed, flour vs. extracted protein).

The results of trials investigating the effects of extracted plant protein on muscle health, revealed that pea, rice and hemp were successful in improving muscle mass, strength and power, given in relatively high doses in young people during training [44,48,55,56]. The outcomes achieved were comparable to those obtained with whey [44,48,55] and soya [56] protein intake, which suggests that pea, rice and hemp isolates might be an effective, vegetarian alternative during training. No other studies were identified in the search, apart from one trial on oat protein, which showed a beneficial effect on muscles during recovery [64]. However, the latter study used maltodextrin as a placebo. It would be interesting to explore whether protective effects of oat protein are greater than those after animal or other plant protein intake. The results of the studies included are in line with a recent review on plant protein and muscle health [77], where authors concluded that although plant protein has the ability to stimulate muscle protein synthesis, some strategies can be considered to improve the anabolic potential of plant protein, like fortifying plant-based protein with certain amino acids, mixing different plant proteins or complementing a plant-based diet with animal protein [77].

Since most studies used isonitrogenous and/or isocaloric designs in terms of intervention treatments, the results show that plant protein are a promising alternative to often less sustainable animal sources. Although no studies on the remaining plant proteins were identified in the search, an interest in these alternative protein sources has been growing and gaining increasing attention over the last few years. Beneficial effects on studied health outcomes were observed for pumpkin [78,79,80], chia seed [81,82], potato [83], buckwheat [84], duckweed [85,86] and sunflower [80]. However, these interesting studies could not be included in the current review as they did not meet the inclusion criteria. The protein used in these interventions were not in a purified preparation form, or were a part of a protein blend, or the effects were studied in conjunction with other nutrient(s) or botanicals with confirmed effects on studied outcomes.

## 5. Industry Perspective—Invited Commentary

### 5.1. Changing Consumer Preferences

**Figure 2:**
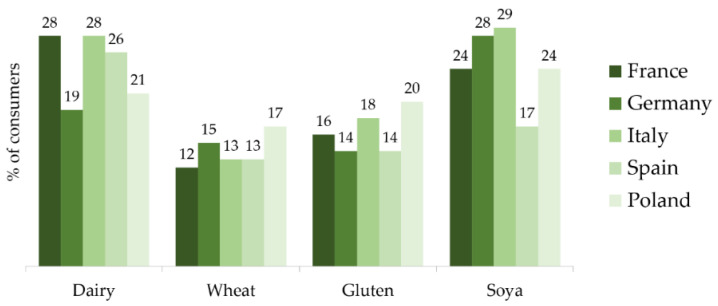
Foods and ingredients avoided by European consumers. (Q4 2018, base: internet users aged 16+, 1000 in each country) [17].

In Western countries, as consumers seek ‘free-from’ products (foods which have had certain ingredients removed, like soya or gluten) (Figure 2) [17]. As a consequence, the demand for alternative sources of proteins, such as legumes (pea, chickpea), other vegetables, cereals such as oats and pseudo-cereals like quinoa and amaranth, is increasing [17].

### 5.2. How Has the Market Responded?

**Figure 3:**
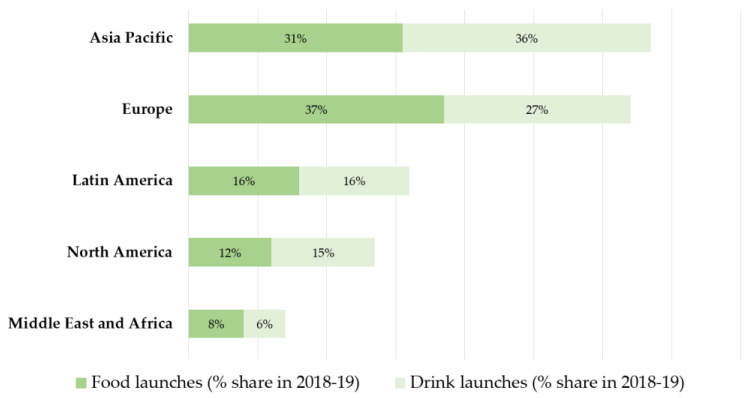
Global food and drink launches that contain added plant protein ingredients [17].

Consumer interest in plant-based diets has led to both an increased innovation in plant-based meat and dairy food and beverage alternatives, and a rise in the development of new plant protein ingredients. Global launches of foods and drinks with added plant protein ingredients are rising, especially in Western markets. In Europe, these new products account for a 37% share in food launches and 27% in new beverages (Figure 3) [17]. The fastest growth of plant proteins has been seen in beverages such as meal replacement drinks and dairy alternatives [87].

Plant protein ingredients take on various roles in these plant-based products [17]. For example, cost-effective plant protein ingredients like wheat gluten are used as a base in breakfast cereals and meat analogue products. Plant protein ingredients are also used to deliver technical properties such as texture, for example in vegan beverages. Finally, plant protein ingredients can enable nutrition claims or improve the nutritional profile, for example, to be labelled ‘high protein’. This is particularly the case for dairy-alternative products, such as yoghurts and dairy-free beverages.

**Figure 4:**
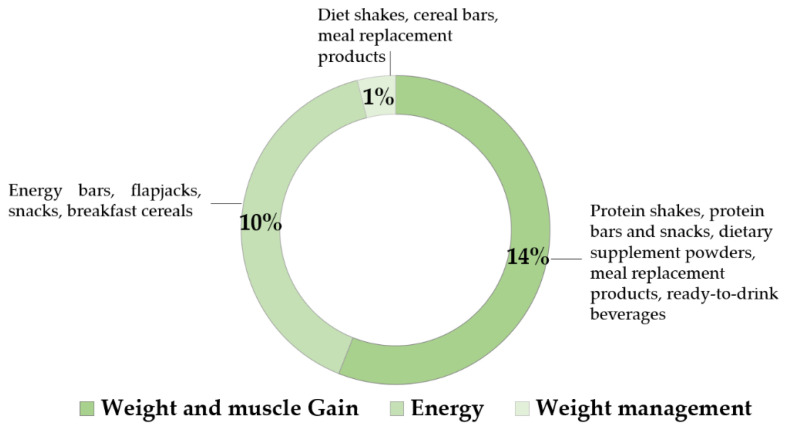
Compound annual growth rate (2015–2019) by on-pack claims that contain added plant protein ingredients, with product examples [87].

Interestingly, global brands choose to communicate the presence of plant proteins in their products in a variety of ways, as shown in Figure 4. Nutrition and health claims on the pack include increased energy, muscle health and better weight management [87].

### 5.3. Innovation in Plant Protein Ingredients Is Changing

**Figure 5:**
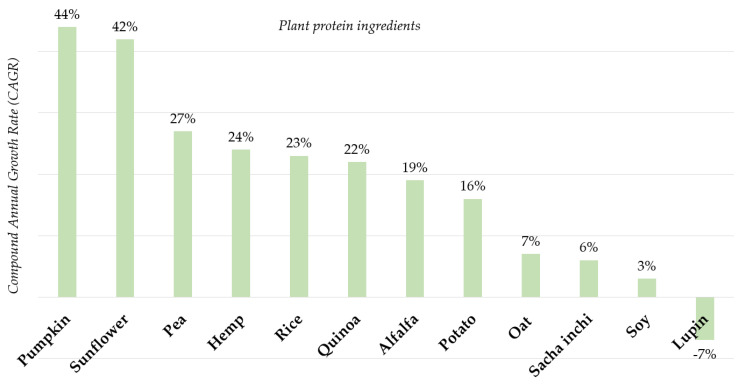
Global compound annual growth rate (2015–2019) in plant protein ingredients: all forms included (isolates, hydrolysates and mildly processed flours) (Mintel GNPD, 2015–2019) [77].

While soya remains the leading global plant protein ingredient, there is a drive for ‘new’ plant protein sources (Figure 5). This has been driven by the notion that soya may not be as environmentally friendly as other alternative plant proteins. Indeed, the sources of proteins that are seeing the biggest growth for product development are pea, sunflower, pumpkin, rice, potato, quinoa and sacha inchi seed proteins [17,87]. Interestingly, the fastest-growing format for plant proteins is isolates (as opposed to concentrates or flours) [87], which may indicate that more refined and isolated forms of plant proteins offer food manufacturers better sensory, taste and nutrition profiles. This can be explained by processing methods used to obtain an isolate, which reduce the off-flavour compounds that originate from phenolic compounds, saponins and oxidation of unsaturated fatty acids. The off-flavour is defined as an unpleasant flavour, including the perception of unpleasant taste, aroma, and other effects, such as astringency [88]. Efficient lipid extraction during the process of purification, use of organic solvents to remove phenolic compounds or isoelectric washing of unprocessed product have been listed among strategies to minimise or eliminate the off-flavour in plant protein ingredients [88].

While improvements in sensory qualities and the stability of plant protein ingredients have been a priority for a number of years, improving the nutrition profile of plant proteins has moved into the limelight more recently. Indeed, protein quality rather than quantity is becoming noticed by consumers [17]. However, it is not clear whether consumers are perceiving protein quality in the context of amino acid composition or as a ‘protein package’ [89], which sees the plant source as a carrier of not only protein but also other health-promotion components, such as dietary fibre, polyphenols, vitamins and polyunsaturated fatty acids). The majority of US consumers (58%) agreed that meat is the most complete source of protein, which may reflect some level of understanding of amino acid composition of protein, and in this context animal proteins are indeed of superior quality. On the other hand, 43% respondents believe that plant-based foods can provide sufficient protein to meet nutritional needs (February 2019 survey, base: 2000 internet users aged 18+) [17]. Perhaps the latter group perceive the product in the context of overall health benefits that may stem from its consumption. Ingredient innovators are responding by exploring ways of improving the digestibility of plant protein, fortifying plant proteins with individual amino acids and blending plant proteins for a more balanced amino acid profile.

### 5.4. Strengths and Limitations

The strengths of this systematic review include registration with PROSPERO prior to data extraction and the use of PICOS format to structure the question [39]. The application of PRISMA guidelines and clearly specified eligibility criteria were used to provide an objective procedure for inclusion and data extraction [38]. The review mapped the existing evidence on concentrated plant proteins and synthesised the results from 26 studies, which was the main goal of this review. Also, the paper includes an industry insight, which is a valuable supplement to the evidence compiled from academic research.

The main limitations refer to the necessity of excluding numerous papers of high relevance to the subject of the review. This was stipulated by the inclusion criteria. The study aim was to include studies in which the studied plant was virtually the only protein source in the tested product. The decisions whether the protocol meets this criterion were made on a case-by case basis, and required subjective assessment of the intervention used. Numerous studies used the concentrate or isolate as an additional protein ingredient, added to other types of protein (e.g., wheat flour in bread, pasta or biscuits recipes). If there is a risk that the health effect could have been (even partially) attributed to other components in the tested product, the study was considered as not suitable for the inclusion. Hence, a large proportion of valuable studies had to be excluded from the final summary. Secondly, although the search strategy was carefully designed it cannot be ruled out that some studies were not picked up during the screening process. Lastly, heterogeneity between the studies in terms of design, dosage, protein carrier and control was low and did not allow direct comparisons to be made and the effect size to be calculated. As a result, this limits the strength of the conclusions drawn in this review and at this stage prevents us from formulating practical recommendations regarding dietary intake.

Through the literature search we have encountered a large body of evidence of plant proteins used as a functional ingredient, usually used in smaller quantities, in bakery products and pasta. Since the aim of this study was to study isolated effects of purified proteins, this aspect has not been explored. It would be interesting to collate the evidence on the effects of concentrated protein on health outcomes as functional ingredients in these products and investigate what dosage or nutritional matrices of food carriers influence each of those outcomes. It would be recommended focusing on each health outcome in a separate review, due to a larger number of studies which used the described approach.

## 6. Conclusions

Multiple studies confirm the health-promoting effect of some extracted plant proteins (pea, lupin, fava bean, rice, oat, hemp and lentil) across glycaemic, appetite, cardiovascular and muscular outcomes when compared to baseline and non-protein controls, with varied effects when compared to other protein treatments (usually milk proteins). Although further research is required, this provides evidence-based support for the need to design functional ingredients that meet both industry and consumer demands. Within the globally emerging flexitarian movement, plant based nutrition has appeared to become the leading dietary trend in the upcoming years. With numerous currently ongoing trials on various plant protein ingredients, it can be expected that more evidence will gradually become available.

The main implication of our work is the current evidence is still not sufficient to formulate explicit dietary recommendations. The effects of plant protein from identified studies were comparable to protein originating from animals. However, in most studies, plant proteins were not superior to animal proteins in terms of measured health outcomes. Still, this is a promising finding, since the desired health effects can be obtained with more sustainable, plant alternatives, with can potentially provide additional health benefits stemming from the plant ‘protein package’ [89]. Hence, more methodologically homogenous research is needed to utilise those and future findings in formulating evidence-based health claims, and translate nutrition-related messages into a consumer-friendly format to enable informed, health-promoting dietary choices.

## Figures and Tables

**Table 1:** Use of PICOS format, as applied to this study.

PICOS Format	Description
Population	Adults ≥18 years old; no restrictions regarding sex or health status
Intervention	Intake of dietary proteins from the following sources: pea, chickpea, lentil, lupin, fava bean, hemp, sunflower, pumpkin, oat, rice, rapeseed, buckwheat, quinoa, duckweed, alfalfa, potato, sacha inchi, mushrooms. No restrictions regarding the dose or intervention duration were applied. Only extracted forms were considered (concentrates, isolates or hydrolysates).
Comparisons	Other protein sources (animal or plant), placebo/control (e.g., water, maltodextrin, other foods with manipulated macronutrient content) or receiving another intervention.
Outcomes	Changes in blood glucose, insulin, satiety hormones and subjective satiety, cardiometabolic risk factors (blood lipids, blood pressure), muscle mass and strength and change in body weight
Study type	Interventional studies; acute or chronic; parallel or crossover design.

**Table 2:** Summary of included studies (*n* = 27).

	Reference	Design	Plant Source	Form	Reported Health Outcomes
1.	Abou-Samra et al. (2011) [45]	Open, single-blind randomised, cross-over	Pea	I	Glycaemia and satiety
2.	Babault et al. (2015) [44]	Double-blind, randomised, placebo-controlled, parallel	Pea	I	Muscle health
3.	Bahr et al. (2013) [46]	Randomised, controlled, cross-over	Lupin	I	Blood lipids, blood pressure, body weight
4.	Bahr et al. (2014) [47]	Randomised, double-blind cross-over	Lupin	I	Blood lipids, blood pressure, body weight
5.	Banaszek et al. (2019) [48]	Randomised, double blind, parallel	Pea	I	Muscle health, body weight
6.	Baum et al. (2017) [49]	Randomised, cross-over	Pea	I	Glycaemia and satiety
7.	Claessens et al. (2007) [50]	Single blind, cross-over	Pea	H	Glycaemia and satiety
8.	Claessens et al. (2009) [51]	Repeated measures with Latin square randomisation, single blind	Pea, rice	H	Glycaemia and satiety
9.	Contaldo et al. (1983) [42]	Cross-over	Fava bean	C	Glycaemia and satiety, blood lipids, body weight
10.	Diepvens et al. (2008) [52]	Randomised, cross over	Pea	H	Glycaemia and satiety
11.	Fabek et al. (2016) [53] *	Two randomised, cross-over, repeated measures	Lentil	C and I	Glycaemia and satiety
12.	Geraedts et al. (2011) [54]	Single blind, randomised, controlled, cross-over	Pea	I	Glycaemia and satiety
13.	Joy et al. (2013) [55]	Randomised, double blind, parallel	Rice	I	Muscle health, body weight
14.	Kaviani et al. (2016) [56] *	Randomised, double blind, parallel	Hemp	C	Muscle health
15.	Lang et al. (1998) [57]	Within-subjects design (two-tail Latin square)	Pea	I	Glycaemia and satiety
16.	Lefranc-Millot et al. (2015) [58] **	Double blind, randomised, placebo control, cross-over	Pea	I	Glycaemia and satiety
17.	Li et al. (2011) [43]	Randomised, double blind, placebo-controlled, cross-over	Pea	H	Blood pressure
18.	Mollard et al. (2014) [40]	Randomised, cross-over, single-blinded	Pea	I	Glycaemia and satiety
19.	Mollard et al. (2017a) [59] *	Repeated measures, cross-over, randomised	Fava bean	C and I	Glycaemia and satiety
20.	Mollard et al. (2017b) [60] *	Repeated measures, cross-over, randomised	Hemp	C	Glycaemia and satiety
21.	Sirtori et al. (2012) [61]	Randomised, double blind, parallel	Pea, lupin	I	Glycaemia and satiety, blood lipids
22.	Smith et al. (2012) [41]	Single blind, randomised, repeated-measures	Pea	I	Glycaemia and satiety
23.	Tan et al. (2018) [62]	Randomised, controlled, cross-over	Oat, rice, pea	C	Glycaemia and satiety, blood lipids
24.	Teunissen-Beekman et al. (2014) [63]	Double blind, six-arm randomised, cross-over	Pea	I	Glycaemia and satiety, blood pressure
25.	Xia et al. (2018) [64]	Randomised, double-blind, placebo-controlled	Oat	I	Muscle health
26.	Weisse et al. (2010) [65]	Randomised, double blind, placebo controlled, parallel	Lupin	I	Glycaemia and satiety, blood lipids

C—concentrate; H—hydrolysate; I—isolate; * Abstracts only (full papers in press or not published); ** conference poster.

**Table 3:** Satiety, glycemic and insulinemic responses to plant protein.

Reference	Protein Source	Population	Intervention		Comparator(s)	Duration	Results
			Dose	Form			
Abou-Samra et al. (2011) [45]	Pea proteins (isolate; 90% protein)	32 males, mean age: 25 ± 4 (E1) and 25 ± 0.6 (E2)	≈20 g	Drink	whey, maltodextrin, casein, egg albumin and water (control)	1 day (acute)	E1: Ad libitum energy intake ↓ after casein and pea preloads compared to water control; Combined satiety score ↑ after casein and pea compared to other preloads; plasma glucose response to ad libitum meal ↓ when whey protein used as a preload (compared to other preloads); E2: cumulative EI (preload + ad libitum meal) ↑ after pea, casein and whey in comparison to water.
Baum et al. (2017) [49]	Pea proteins	23 females, 10 males, mean age 23.5	41 g	Breakfast beverage	Whey protein isolate	1 day (acute)	No difference in postprandial appetite response between pea and whey protein.
Claessens et al. (2007) [50]	Pea protein hydrolysate (PPH) with a CHO afterload	8 males, mean age 28.5 ± 3.6	0.4 g protein/kg of body weight	Drink	Soy protein hydrolysate (SPH), maltodextrin (control)	5 trials, each 1 day (acute)	Pea protein with a CHO afterload induced ↑ insulin and glucagon responses (area under the curve) than SPH with CHO afterload.
Claessens et al. (2009) [51]	Pea protein hydrolysate (PPH) and rice protein hydrolysates (RPH)	8 males, mean age 32 ± 13.8	0.2 g hydrolysate/kg of body weight	Drink	Rice, soy, gluten, whey, egg hydrolysates, and maltodextrin (control)	7 trials, each 1 day (acute)	No difference in glucose and insulin response at peak (delta 30) between hydrolysates. Glucagon response ↑ only for soy (when compared to gluten). Glucagon AUCs differed only between gluten and egg.
Contaldo et al. (1983) [42]	Fava bean concentrate	4 females, 4 males, age 43.4	50 g (F); 70 g(M) daily	Integrated into a diet	Egg whites	18 days	Fasting BG ↓ after faba bean, compared to baseline. Insulin unchanged in both diets.
Diepvens et al. (2008) [52]	Pea protein hydrolysate (PPH)	20 females, 19 males, mean age 42.3 ± 13.8	15 g	Shake	Whey protein (WP), and a blend of WP + PPH, and milk protein (MP, casein + WP) (control)	1 day in E1 (4 h) and E2 (7 h)	PPH induced ↓ hunger and ↓ desire to eat compared to MP or WP + PPH. A longer intermeal interval and ↑ satiety index after PPH. Both PPH and WP lead to ↑ satiety (E2) and fullness (E2) compared to MP and WP + PPH. GLP-1 and CCK was ↑ after MP, than in four other shakes. No effect on EI was seen.
Fabek et al. (2016) [53] *Abstract only (FASEB)*	Lentil protein concentrate (LPC, 55% protein) and isolate (LPI, 75% protein)	48 males, young (age not specified)	20 g of LPC (55% protein) or LPI (75% protein)	Added to tomato soup	Lentil fibre, lentil starch and tomato soup alone (control)	1 day (acute)	Ad libitum meal after 30 min: only LPI and LPC induced ↓ postprandial glycemia without increasing insulin, and ↓ subjective appetite, compared to the control. Ad libitum meal after 120 min: only lentil starch resulted in ↓ pre-and post-meal subjective appetite, but higher blood glucose than control.
Geraedts et al. (2011) [54]	Pea protein	20 males, mean age: 25 ± 2 (lean), 41 ± 6 (obese)	250 mg/kg of body weight	Drink (oral or intraduodenal admin.)	Water (placebo)	1 day (acute) on 4 occasions	Some changes observed in terms of hunger, fullness, CCK, PYY and food intake, with differences regarding method of protein administration (orally vs. intraduodenal) and nutrition status (lean vs. obese). No difference in satiety and ghrelin level.
Lang et al. (1998) [57]	Pea protein isolate	12 males, mean age 22.6 ± 0.6	45.3 g ± 1.2	Protein manipulated meal	Egg albumin, casein, gelatin, soy protein and wheat gluten	1 day (acute) on 6 occasions	No effect of the type of protein on satiety, on 24-h energy or macronutrient intakes, or on postprandial plasma glucose and insulin concentrations.
Lefranc-Millot et al. (2015) [58] *Conference poster*	Pea protein isolate	22 females, 11 males, age range 18–65	15 g and 30 g	soup	Whey protein and no protein soup (control)	1 day (acute)	Both PP and WP induced ↓ caloric intake, compared to control. Favorable modulation of glucose, insulin and and some satiety hormones, but mainly when compared to control, rather than other protein treatments.
Mollard et al. (2014) [40]	Pea protein (82% protein)	15 males, mean age 21.5 ± 1.0	10 g	Added to meal	Pea hull fibre, pea hull fibre + pea protein, canned yellow peas, noodles with tomato sauce (control)	1 day (acute), with 1 treatment (or control) per week	No effect of treatment on food intake, pre and post pizza subjective appetite. In terms of PP, between treatments difference was observed only in comparison to fibre (↓ pre-meal BG AUC and ↓ BG cumulative AUC).
Mollard et al. (2017a) [59] *Abstract*	Fava bean concentrate (FBC) and isolate (FBI)	15 males, young (age not specified)	32 g FBC 32 g FBI	Smoothies	whole FB flour, high-starch FB flour, corn maltodextrin (control)	1 day (acute)	All flours had favourable effect on pre-meal glucose and iAUC, in comparison to control. Between treatment differences were observed for FBC and FBI (↓ blood post meal glucose iAUC when compared to FB starch).
Mollard et al. (2017b) [60] *Abstract*	Hemp protein concentrate (HPC)	16 adults, young (age not specified)	20 g and 40 g	Fruit shakes	Soybean concentrate (SBC), carbohydrate (control)	1 day (acute)	Favourable effects (dose-dependent) of all protein treatments (soy and hemp) on glucose and insulin; in general no significant differences between protein type. In addition, HPC (40 g) led to ↑ glucose and insulin responses following a fixed meal 60 min after protein ingestion.
Sirtori et al. (2012) [61]	Pea protein isolate (PPI) and cellulose/oat fibre/pectin, Lupin protein isolate (LPI) and cellulose	93 females, 82 males; mean age 53.9	Two bars a day (34.6 g day)	Bars	Casein and cellulose, casein and oat fibre, casein and pectin	4 wk	At 4 wk, glucose ↓ only after pea protein and oat fibre, in comparison to baseline. Insulin ↓ after casein and cellulose, casein and pectin, pea protein and oat fibre, compared to baseline
Smith et al. (2012) [41]	Yellow pea protein	E1: 19 males, mean age 23.2 ± 0.5 E2: 20 males, mean age 22.3 ± 0.5	Two treatments: 10 and 20 g	Added to tomato soup	Tomato soup with 10 and 20 g fibre, tomato soup with no added fibre or protein (control)	1 day (acute study)	E1: 20 g protein led to ↓ food intake (FI) than control and all other treatments and ↓ cumulative FI compared to 10 g fibre. Both protein doses stipulated ↓ pre-meal glucose (0–30 min) compared to control; only 20 g protein suppressed mean post-meal BG (50–120 min). No effect of on pre-meal or post-meal appetite. E2: no effect of treatment on FI, CFI, or pre-or post-meal BG or appetite.
Tan et al. (2018) [62]	Oat, rice and pea concentrates	20 males, mean age 26 ± 5	24 g	Chocolate beverage	Chocolate beverage without protein	1 day (acute)	Insulin iAUC was ↑ after oat and pea, but not rice ingestions, in comparison to control. No sig. differences in GIP and GLP-1. Sig. effect of time on hunger, fullness, desire to eat, but with no sig. effect of treatment.
Teunissen-Beekman et al. (2014) [63]	Pea protein isolate	31 males, 17 females, mean age 58 ± 1	0.6 g of protein/kg of body weight	Drink	Milk protein isolate, an egg-white protein isolate, blend of protein isolates, maltodextrin and sucrose	1 day (acute study)	The ingestion of all proteins resulted in ↓ plasma glucose concentrations and ↑ insulin (iAUC). No differences in the postprandial BG responses to the ingestion of all the three proteins. Insulin ↓ after egg-white (than milk) after 1–2 h, and ↑ after milk than pea (at 4 h). Glucagon ↑ after all protein, but lowest after egg-white, than other proteins (at 1–3 h). At 1–2 h, highest glucagon after pea protein. GLP-1 highest after all 3 protein, but ↓ after egg-white than of other protein (at 2 h), and ↑ after egg-white than pea (at 4 h).
Weisse et al. (2010) [65]	Lupin isolate	23 females, 20 males; mean age 43.9 ± 11.8	17.5 g twice a daily	Bars	Casein	6 weeks	No changes in plasma glucose from baseline or between the interventions.

**Table 4:** Blood lipids.

Reference	Protein Source	Population	Intervention		Comparator(s)	Duration	Results
			Dose	Form			
Bahr et al. (2013) [46]	Lupin protein isolate (LPI)	18 females, 15 males, mean age 49.7 ± 12.8	25 g/daily	Drink	Milk protein isolate (MPI)	8 weeks	At wk 4 ↓ in LDL and LDL:HDL in LPI group and ↓ LDL in MPI group. The only difference between treatments in HDL at wk4 (↑ in LPI and ↓ in MPI). After 8 wks ↑ in triglicerides (LPI) and no changes from baseline in MPI group.
Bahr et al. (2014) [47]	Lupin protein isolate (LP)	41 females, 31 males, 18–80 y	25 g/day	Integrated into a mixed diet	Milk protein (MP), MP + 1.6 g/d arginine (MPA)	28 days	Compared to baseline, total cholesterol, LDL, HDL was ↓ in LP and MPA groups. Triglycerides were only ↓ in LP group. Between treatments differences only for total cholesterol (↓ in MPA vs. MP) and LDL (↓ in LP vs. MP). The relative changes in total and LDL cholesterol were significantly greater for subjects with severe hypercholesterolemia than those with moderate hypercholesterolemia
Contaldo et al. (1983) [42]	Fava bean concentrate	4 females, 4 males, age 43.4	50 g (F); 70 g(M) daily	Integrated into a diet	Egg whites	18 days	Total cholesterol ↓ in both treatments, but LDL was significantly ↓ only in egg-white diet. HDL decreased only on the fava bean diet. Serum total and VLDL triglyceride showed no changes.
Sirtori et al. (2012) [61]	Pea protein isolate (PPI) and cellulose/oat fibre/pectin, Lupin protein isolate (LPI) and cellulose	93 females, 82 males; mean age 53.9	Two bars a day (34.6 g day)	Bars	Casein and cellulose, casein and oat fibre, casein and pectin	4 wk	Lupin protein and cellulose, casein and pectin, pea protein and oat fibre and pea protein and pectin resulted in reduction of total cholesterol. Decrease in LDL only after pea protein + pectin and pea protein + oat fibre. No changes in triglicerides or HDL.
Tan et al. (2018) [62]	Oat, rice and pea concentrates	20 males, mean age 26 ± 5	24 g	Chocolate beverage	Chocolate beverage without protein	1 day (acute)	Serum triglyceride excursions and iAUC did not differ between all test beverages.
Weisse et al. (2010) [65]	Lupin isolate	23 females, 20 males; mean age 43.9 ± 11.8	17.5 g twice a daily	Bars	Casein	6 weeks	Both treatments resulted in ↓ plasma cholesterol from baseline. In addition, LP (reduction of LDL), while casein (reduction of HDL and triglicerides), from baseline. Between the groups difference only for LDL:HDL ratio (↑ in casein, and ↓ in LP group)

**Table 5:** Blood pressure.

Reference	Protein Source	Population	Intervention		Comparator(s)	Duration	Results
			Dose	Form			
Bahr et al. (2013) [46]	Lupin protein isolate (LPI)	18 females, 15 males, mean age 49.7 ± 12.8	25 g/daily	Drink	Milk protein isolate (MPI)	8 weeks	At 8 wks, ↓ in SBP and DBP in LPI group, and only SBP in MPI (from baseline) but no differences between treatments.
Bahr et al. (2014) [47]	Lupin protein isolate (LP)	41 females, 31 males, 18–80 y	25 g/day	Integrated into a mixed diet	Milk protein (MP), MP + 1.6 g/d arginine (MPA)	28 days	No changes in SBP or DBP from baseline or between the treatments
Li et al. (2011) [43]	Pea protein hydrolysate (peptides)	4 females, 3 males; age 30–55 y	Two treatments: 1.5 and 3 g (spread over 3 meals)	Orange juice	Orange juice (placebo)	3 weeks	In comparison to placebo, a ↓ in SBP after 2 and 3 weeks supplementation of PPH at 3 g/day dose.
Teunissen-Beekman et al. (2014) [63]	Pea protein	17 females, 31 males; mean age: 58 ± 1	0.6 g of protein/kg of body weight	Drink	Milk protein isolate, an egg-white protein isolate, mix of protein isolates	1 day (acute study)	Egg-white protein resulted in ↑ in SBP, while pea protein ↓ DBP. Postprandial BP levels were ↓ after maltodextrin than after protein mix and sucrose meals.

**Table 6:** Muscle health.

Reference	Protein Source	Physical Activity	Population	Intervention		Comparator(s)	Duration	Results
				Dose	Form			
Babault et al. (2015) [44]	Pea proteins	12-week resistant training	161 males, mean age: 22 ± 3.5	25 g/twice a day	Drink	Whey and placebo	12 weeks	Thickness increased from baseline in all groups. Highest increase in pea group at 12 wks, compared to placebo. No differences between the groups in terms of muscle circumference at week 12.
Banaszek et al. (2019) [48]	Pea proteins	8-week high-intensity-functional training (HIFT)	7 females and 8 males, mean age: 38.6 ± 12.7 (M), 38.9 ± 10.9 (F)	24 g/twice a day	Drink	Whey protein, no control	8 weeks	Increase in muscle strength, squats and deadlift, from baseline, but no differences between the interventions.
Joy et al. (2013) [55]	Rice protein isolate (RPI)	8-week resistance training	24 males, mean age: 21.3 ± 1.9	48 g	Drink	Whey protein isolate (WPI), no control	8 weeks	Increase in strength, power and thickness after 8 weeks from the baseline for both RPI and WPI, with no differences between the groups.
Kaviani et al. (2016) [56]	Hemp protein (HP) powder	8-week resistance training	28 males, 12 females (age not provided)	60 g powder (40 g protein)	Not specified	Soy protein	8 weeks	Increased strength and muscle thickness in females, but not males, after HP powder, compared to soy group.
Xia et al. (2018) [64]	Oat isolate	Exhaustive downhill running	16 males, mean age: 19.7 ± 1.1	12.5 g/twice a day (14 days before exercise and 4 days thereafter)	Drink	Maltodextrin	19 days	Compared to placebo, oat protein ↓ inflammatory markers, muscle soreness (VAS) score, and lessen the loss of function associated with damaging exercise.

**Table 7:** Body weight.

Reference	Protein Source	Population	Intervention		Comparator(s)	Duration	Results
			Dose	Form			
Bahr et al. (2013) [46]	Lupin protein isolate (LPI)	18 females, 15 males, mean age 49.7 ± 12.8	25 g/daily	Drink	Milk protein isolate (MPI)	8 weeks	At 8 wks, slight ↑ in body weight, BMI and body fat in LPI and MPI group, compared to baseline, but no differences between intervention
Bahr et al. (2014) [47]	Lupin protein isolate (LP)	41 females, 31 males, 18 −80 y	25 g/day	Integrated into a mixed diet	Milk protein (MP), MP + 1.6 g/d arginine (MPA)	28 days	At 28 days, increase in WC in MP group, compared to LP group. No changes in body weight, BMI, or body fat in comparison to baseline and between the treatments.
Banaszek et al. (2019) [48]	Pea proteins + PA	7 females and 8 males, mean age: 38.6 ± 12.7 (M), 38.9 ± 10.9 (F)	24 g/twice a day	Drink	Whey protein, no control	8 wk	No change in body mass or body fat from baseline and no differences between the interventions.
Contaldo et al. (1983) [42]	Fava bean concentrate	4 females, 4 males, age 43.4	50 g (F); 70 g(M) daily	Integrated into a diet	Egg whites	18 days	No changes in body weight, except in one patient
Joy et al. (2013) [55]	Rice protein isolate + PA	24 males, mean age: 21.3 ± 1.9	48 g	Drink	Whey protein isolate	48 h (Phase 1) 8 wk (Phase 2)	↑ in lean body mass ↓ of body fat in both interventions at 8 weeks, with no differences between the interventions;

## Supplementary Materials

The following are available online at https://www.mdpi.com/2072-6643/12/8/2291/s1: Search String (PubMed).
