# Supplementary material for: Exploring Health-Promoting Attributes of Plant Proteins as a Functional Ingredient for the Food Sector: A Systematic Review of Human Interventional Studies
Source: Nutrients. 2020 Jul 30;12(8):2291. doi: 10.3390/nu12082291 (PMC7468935; doi:10.3390/nu12082291)
Supplement: Supplementary file 1 [file nutrients-12-02291-s001.pdf]

Search strategy (PubMED example)

#1: "diet, food, and nutrition"[MeSH Terms]

#2: "grain proteins"[MeSH Terms] OR "seed storage proteins"[MeSH Terms] OR "plant proteins, dietary"[MeSH Terms] OR "dietary proteins"[MeSH Terms] OR "protein"[Title/Abstract] OR "proteins"[Title/Abstract] OR protein's[Title/Abstract]

#3: "peas"[MeSH Terms] OR peas[Title/Abstract] OR pea[Title/Abstract] OR "pisum sativum"[Title/Abstract] OR "lens plant"[MeSH Terms] OR "lens plant*"[Title/Abstract] OR lentil[Title/Abstract] OR lentils[Title/Abstract] OR "lens culinaris"[Title/Abstract] OR "cicer"[MeSH Terms] OR cicer[Title/Abstract] OR chickpea[Title/Abstract] OR chickpea'[Title/Abstract] OR chickpea's[Title/Abstract] OR chickpeas[Title/Abstract] OR chickpeas'[Title/Abstract] OR garbanzo[Title/Abstract] OR "vicia faba"[MeSH Terms] OR "vicia faba"[Title/Abstract] OR "faba bean"[Title/Abstract] OR "faba beans"[Title/Abstract] OR "fava bean"[Title/Abstract] OR "fava beans"[Title/Abstract] OR "broad bean"[Title/Abstract] OR "broad beans"[Title/Abstract] OR "helianthus"[MeSH Terms] OR helianthus[Title/Abstract] OR sunflower[Title/Abstract] OR sunflower'[Title/Abstract] OR sunflower's[Title/Abstract] OR sunflowers[Title/Abstract] OR sunflowerseed[Title/Abstract] OR sunflowerseeds[Title/Abstract] OR "cucurbita"[MeSH Terms] OR pumpkin[Title/Abstract] OR pumpkin'[Title/Abstract] OR pumpkin's[Title/Abstract] OR pumpkins[Title/Abstract] OR pumpkinseed[Title/Abstract] OR pumpkinseed's[Title/Abstract] OR pumpkinseeds[Title/Abstract] OR "fagopyrum"[MeSH Terms] OR fagopyrum[Title/Abstract] OR buckwheat[Title/Abstract] OR "chenopodium quinoa"[MeSH Terms] OR "chenopodium quinoa"[Title/Abstract] OR quinoa[Title/Abstract] OR "solanum tuberosum"[MeSH Terms] OR Potato[Title/Abstract] OR potatoes[Title/Abstract] OR "solanum tuberosum"[Title/Abstract] OR mushroom[Title/Abstract] OR mushrooms[Title/Abstract] OR "oryza"[MeSH Terms] OR "oryza sativa"[Title/Abstract] OR rice[Title/Abstract] OR "lupinus"[MeSH Terms] OR lupinus[Title/Abstract] OR lupin[Title/Abstract] OR lupins[Title/Abstract] OR "avena"[MeSH Terms] OR "avena sativa"[Title/Abstract] OR oat[Title/Abstract] OR oats[Title/Abstract] OR duckweed[Title/Abstract] OR wolffia[Title/Abstract] OR mankai[Title/Abstract] OR lemnaceae[Title/Abstract] OR lemna[Title/Abstract] OR lemnoideae[Title/Abstract] OR "water lentil*"[Title/Abstract] OR "medicago sativa"[MeSH Terms] OR "medicago sativa"[Title/Abstract] OR alfalfa[Title/Abstract] OR plukenetia volubilis[Title/Abstract] OR sacha inchi[Title/Abstract] OR "inca peanut*" [Title/Abstract] OR cannabis[MeSH Terms] OR Hemp[Title/Abstract] OR cannabis[Title/Abstract] OR rapeseed*[Title/Abstract] OR Brassica[MeSH Terms] OR brassica[Title/Abstract] OR rape seed* OR canola)

#4: "appetite"[MeSH Terms] OR appetite[Title/Abstract] OR "satiety response"[MeSH Terms] OR satiety[Title/Abstract] OR satiation[Title/Abstract] OR "hunger"[MeSH Terms] OR hunger[Title/Abstract] OR fullness[Title/Abstract] OR "cholecystokinin"[MeSH Terms] OR cholecystokinin[Title/Abstract] OR CCK[Title/Abstract] OR "leptin"[MeSH Terms] OR leptin[Title/Abstract] OR "glucagon like peptide 1"[MeSH Terms] OR "glucagon-like peptide-1"[Title/Abstract] OR GLP-1[Title/Abstract] OR "peptide yy"[MeSH Terms] OR "peptide yy"[Title/Abstract] OR "ghrelin"[MeSH Terms] OR ghrelin[Title/Abstract] OR "visual analog scale"[MeSH Terms] OR "visual analog scale"[Title/Abstract] OR "postprandial glycemia"[Title/Abstract] OR "postprandial glycaemia"[Title/Abstract] OR "visual analogue scale"[Title/Abstract] OR "body weight"[MeSH Terms] OR "body weight"[Title/Abstract] OR "body weight changes"[Title/Abstract] OR "body weight changes"[MeSH Terms] OR "body mass index"[MeSH Terms] OR "body mass index"[Title/Abstract] OR "fat free mass"[Title/Abstract] OR "fat-free mass"[Title/Abstract] OR "weight loss"[MeSH Terms] OR "weight loss"[Title/Abstract] OR "weight reduction"[Title/Abstract] OR "body composition"[MeSH Terms] OR "body composition"[Title/Abstract] OR "waist circumference"[MeSH Terms] OR "waist circumference"[Title/Abstract] OR "abdominal fat"[MeSH Terms] OR "abdominal fat"[Title/Abstract] OR "visceral fat"[Title/Abstract] OR "body fat"[Title/Abstract] OR "abdominal visceral fat"[Title/Abstract] OR "adipose tissue"[MeSH Terms] OR "adipose tissue"[Title/Abstract] OR "adiposity"[MeSH Terms] OR adiposity[Title/Abstract] OR "overweight"[MeSH Terms] OR overweight[Title/Abstract] OR "obesity"[MeSH Terms] OR obesity[Title/Abstract] OR obese[Title/Abstract] OR "diabetes mellitus"[MeSH Terms] OR diabetes[Title/Abstract] OR diabetic[Title/Abstract] OR "hyperglycemia"[MeSH Terms] OR hyperglycemia[Title/Abstract] OR

hyperglycemic[Title/Abstract] OR "blood glucose"[MeSH Terms] OR "blood glucose"[Title/Abstract] OR "insulin"[MeSH Terms] OR insulin[Title/Abstract] OR "insulin resistance"[MeSH Terms] OR "postprandial glucose"[Title/Abstract] OR "postprandial insulin"[Title/Abstract] OR "fasting glucose"[Title/Abstract] OR f-glc[Title/Abstract] OR "homeostasis model assessment-insulin resistance"[Title/Abstract] OR HOMA-IR[Title/Abstract] OR HOMA-IS[Title/Abstract] OR "glucose tolerance test"[MeSH Terms] OR "glucose tolerance test"[Title/Abstract] OR OGTT[Title/Abstract] OR "AUC glucose"[Title/Abstract] OR "iAUC glucose"[Title/Abstract] OR "AUC insulin"[Title/Abstract] OR "iAUC insulin"[Title/Abstract] OR "cardiovascular diseases"[MeSH Terms] OR "cardiovascular diseases"[Title/Abstract] OR "cardiovascular disease"[Title/Abstract] OR "hypercholesterolemia"[MeSH Terms] OR hypercholesterolemia[Title/Abstract] OR hypercholesterolemic[Title/Abstract] OR "hyperlipidemias"[MeSH Terms] OR hyperlipidemia[Title/Abstract] OR hyperlipidemias[Title/Abstract] OR hyperlipidemic[Title/Abstract] OR "dyslipidemias"[MeSH Terms] OR dyslipidemia[Title/Abstract] OR dyslipidemias[Title/Abstract] OR dyslipidemic[Title/Abstract] OR "blood lipid"[Title/Abstract] OR "blood lipids"[Title/Abstract] OR "lipid profile"[Title/Abstract] OR "cholesterol"[MeSH Terms] OR cholesterol[Title/Abstract] OR "triglycerides"[MeSH Terms] OR trigliceride[Title/Abstract] OR triglicerides[Title/Abstract] OR HDL[Title/Abstract] OR LDL[Title/Abstract] OR "metabolic syndrome"[MeSH Terms] OR "metabolic syndrome"[Title/Abstract] OR "blood pressure"[MeSH Terms] OR "blood pressure"[All Fields] OR systolic[Title/Abstract] OR diastolic[Title/Abstract] OR "hypertension"[MeSH Terms] OR hypertension[Title/Abstract] OR hypertensive[Title/Abstract] OR antihypertensive[Title/Abstract] OR "muscles"[MeSH Terms] OR muscle[Title/Abstract] OR muscles[Title/Abstract])

#5 ((randomized controlled trial[pt] OR controlled clinical trial[pt] OR (randomized[tiab] OR randomised[tiab]) OR placebo[tiab] OR "drug therapy"[Subheading] OR randomly[tiab] OR trial[tiab] OR groups[tiab]))) NOT ("animals"[MeSH Terms] NOT "humans"[MeSH Terms])

#6: #1 AND #2 AND #3 AND #4 AND #5
